# DNase1: No Association with Crohn's Disease in a New Zealand Population

**DOI:** 10.5402/2012/826323

**Published:** 2012-06-04

**Authors:** Angharad R. Morgan, Wen-Jiun Lam, Dug-Yeo Han, Alan G. Fraser, Lynnette R. Ferguson

**Affiliations:** ^1^Discipline of Nutrition, Faculty of Medical and Health Sciences (FMHS), The University of Auckland, 85 Park Road, Grafton, 1142 Auckland, New Zealand; ^2^Nutrigenomics New Zealand, New Zealand; ^3^Department of Medicine, Faculty of Medical and Health Sciences (FMHS), The University of Auckland, 85 Park Road, Grafton, 1142 Auckland, New Zealand

## Abstract

DNase1 has been implicated in a number of immune disorders and is an excellent candidate gene for Crohn's disease (CD). We investigated whether DNase1 SNPs rs1053874 and rs8176938 were associated with CD in a well-characterized New Zealand dataset consisting of 447 cases and 716 controls. Furthermore, we measured serum DNase1 activity levels in a number of CD patients and controls. We did not find any evidence of association for either DNase1 genetic variation or DNase1 activity levels with CD. The lack of association indicates that DNase1 does not play a significant role in predisposing to CD in the New Zealand population.

## 1. Introduction

Deoxyribonuclease 1 (DNase1) is an endonuclease that facilitates chromatin breakdown. Deficiency in DNase1 and the resulting difficulty of removing DNA from nuclear antigens promote susceptibility to immune disorders such as systemic lupus erythematosus (SLE) [1, 2], Sjogren's disease [3], and thyroid autoimmunity [4]. A recent report has also provided some evidence for a role in Crohn's disease (CD) [5].

Genetic variation in DNase1 has been associated with SLE [6]. Studies of associations between DNase1 genetic variants and CD have not been reported to date.

We investigated whether DNase1 SNPs rs1053874 and rs8176938 were associated with CD in a well-characterized case-control New Zealand dataset. Furthermore, we measured serum DNase1 activity levels in a number of CD patients and controls.

## 2. Methods

### 2.1. Samples

A total of 1163 subjects from New Zealand were included in the study: 447 cases and 716 controls. All participants self-reported European ancestry.

Clinical records were analysed to confirm diagnosis, and IBD status was defined using standard diagnostic criteria [7]. Cases were phenotyped according to the Montreal classification systems.

Participants consented to collection of peripheral blood or a buccal swab for DNA extraction and genotyping, and DNA was extracted from the blood/buccal samples using Qiagen's DNA extraction kit and following the manufacturer's instructions.

The study was conducted under ethical protocol MEC/04/12/011, authorised through the New Zealand Multiregion Human Ethics Committee. All study subjects gave informed consent.

### 2.2. Genotyping

We searched for SNPs in the DNase1 gene in the hapmap database and identified 2 common (MAF > 5%) SNPs: rs1053874 and rs8176938. We genotyped these SNPs using the ABI TaqMan MGB diallelic discrimination system and predesigned assays on demand (Applied Biosystems, Melbourne, Australia). The polymerase chain reaction amplification was performed using the ABI Prism 7900 HT sequence-detector machine using the following conditions: 10 minutes at 95°C, followed by 40 cycles at 92°C for 15 seconds and 60°C for 1 minute. The allelic discrimination results were determined after the amplification by performing an endpoint read.

All sample plates contained cases, controls, blanks, hapmap, and duplicate samples. Quality control measures included independent double genotyping, blind to sample identity, and comparison of our hapmap sample genotypes to those in the hapmap database (http://www.hapmap.org/).

### 2.3. Measurement of DNase1 Activity Levels

DNase1 activity levels were measured in the serum of 213 CD patients and 209 controls using a commercially available validated enzyme-linked immunosorbent assay (ELISA) kit (Orgentec, Germany), and the protocol was followed as described by the manufacturer. All samples were analyzed in duplicate. Activity levels were determined from a standard curve using a 4-parameter-fit with lin-log coordinates for optical density and concentration.

### 2.4. Statistical Analysis

SNPs were tested for deviation from HWE in both cases and controls using a chi-square goodness-of-fit test. To determine if there were differences between cases and controls, genotype and allele frequencies for each SNP were analyzed by 2 × 3 and 2 × 2 Chi-square tables, respectively.

Genotype and phenotype associations were assessed by comparing allele frequencies between controls and patient subgroups defined using the clinical characteristics. These analyses were carried out using R (R: a language and environment for statistical computing, R Foundation for Statistical Computing, Vienna, Austria. ISBN 3-900051-07-0, URL http://www.r-project.org/) and SAS (V9.1 SAS Institute., Cary, NC, USA).

Across-group comparisons of serum DNase 1 activity were tested using a generalized linear model for mean differences.

## 3. Results

### 3.1. Comparison of DNase1 Genotypes in CD Patients and Healthy Controls

Both SNPs were in Hardy-Weinberg equilibrium. Neither was found to be associated with CD (Table 1). We undertook a phenotype analysis, and there were no associations with any of the different clinical characteristics of CD (Table 2).

### 3.2. Comparison of DNase1 Activity Levels in CD Patients and Healthy Controls

DNase1 activity levels were measured in the serum. Activity in the controls ranged from 43 to 100%. Activity in the cases ranged from 39 to 100%. The mean serum DNase1 activity for CD patients was 80% and that for the controls was also 80%. Thus, there was no difference in activity levels between cases and controls. We also compared DNase1 activity levels across the different CD phenotypes, but there were no significant findings (Table 3). Neither there were any differences when comparing by gender.

## 4. Discussion

DNase1 is known to be involved in a number of immune disorders, but research into its role in CD has been rare. There have been no previous studies investigating associations between genetic variation in DNase1 and CD, and only one published study examined DNase1 activity levels in CD.

We were unable to replicate the association reported in previous research in which DNase1 activity in CD patients was significantly lower than in healthy individuals [5]. In our study there was no difference in the DNase1 activity levels. All of the CD patients in the previous study had active severe disease. Our sample set was larger and contained a range of different phenotypes but when we stratify by these different phenotypes, including severity, we were still unable to see any associations with DNase1. Nonreproducibility does not necessarily demonstrate that the original association reported was spurious and should not discourage further research in this promising field. True associations may not replicate across different data sets for a number of reasons.

As well as investigating DNase1 activity levels, we also undertook an analysis of genetic variation in DNase1 and compared the genotype and allele frequencies of 2 SNPs in CD patients and controls. Genetic variation in DNase1 has previously been associated with SLE [6] but in the study we report here we were not able to find any evidence for association between DNase1 SNPs and CD. Data from a recent CD genomewide meta-analysis [8] also does not provide support for DNase1 being associated with CD: SNP rs1053874 was included in the analysis, and the allele frequencies did not differ between cases and controls.

To summarise, although DNase1 is a good candidate for CD, in the present study we failed to detect any significant associations. The absence of association between DNase1 and CD indicates that DNase1 does not play a significant role in predisposing to CD in the New Zealand population.

## Figures and Tables

**Table 1:** Genotype and allele counts (and frequencies) in CD patients and in controls.

SNP		Case	Control	*P*		Case	Control	*P*
rs1053874	A/A	40 (9.0)	65 (9.2)	0.56	A	262 (29.5)	442 (31.2)	0.40
	A/G	182 (41.0)	312 (44.0)		G	626 (70.5)	976 (68.8)	
	G/G	222 (50.0)	332 (46.8)					

rs8176938	C/C	410 (91.7)	644 (91.2)	0.83	C	855 (95.6)	1348 (95.5)	0.85
	C/G	35 (7.8)	60 (8.5)		G	39 (4.4)	64 (4.5)	
	G/G	2 (0.4)	2 (0.3)					

**Table 2:** Relationships between SNPs and subphenotypes.

		rs1053874 (tested allele: G)		rs8176938 (tested allele: C)	
		OR (95% CI)	*P*	OR (95% CI)	*P*
Age at diagnosis	<17	0.86 (0.54–1.36)	0.509	1.45 (0.45–4.60)	0.533
	17 to 40	1.02 (0.81–1.28)	0.900	1.36 (0.80–2.33)	0.259
	40<	1.08 (0.72–1.62)	0.707	0.86 (0.39–1.89)	0.712

CD behaviour	inflammatory	1.12 (0.88–1.44)	0.361	1.27 (0.72–2.22)	0.411
	stricturing	0.78 (0.58–1.06)	0.113	1.01 (0.53–1.95)	0.969
	penetrating	1.22 (0.74–2.00)	0.433	2.20 (0.53–9.10)	0.275

CD location	Ileal	0.96 (0.72–1.29)	0.802	0.86 (0.49–1.52)	0.605
	Colonic	1.11 (0.82–1.51)	0.500	1.62 (0.74–3.54)	0.229
	Ileocolonic	0.95 (0.69–1.30)	0.726	1.75 (0.75–4.10)	0.195

Other IBD family	Y	0.89 (0.54–1.48)	0.651	1.20 (0.37–3.89)	0.759
Bowel resection	Y	0.91 (0.68–1.21)	0.517	1.43 (0.70–2.91)	0.322
EIM	Y	0.89 (0.61–1.32)	0.568	0.90 (0.41–1.98)	0.799
Perianal disease	Y	1.33 (0.84–2.10)	0.226	—	
Severity	Y	1.00 (0.69–1.47)	0.982	1.19 (0.51–2.78)	0.695
Smoking	Y	1.08 (0.74–1.58)	0.677	0.95 (0.34–2.61)	0.916

**Table 3:** DNase1 activity by subphenotype.

		Estimate (95% CI)	*P*
Age at diagnosis	<17	−0.23 (−4.09–3.64)	0.908
	17 to 40	1.51 (−0.75–3.78)	0.190
	40<	−2.37 (−6.39–1.65)	0.247

CD behaviour	inflammatory	−0.16 (−2.66–2.35)	0.903
	stricturing	1.96 (−1.01–4.93)	0.195
	penetrating	0.88 (−3.32–5.07)	0.681

CD location	Ileal	0.10 (−2.76–2.97)	0.943
	Colonic	1.29 (−1.61–4.19)	0.382
	Ileocolonic	0.38 (−2.69–3.44)	0.809

Other IBD family	Y	0.50 (−4.56–5.57)	0.846
Bowel resection	Y	1.38 (−1.47–4.23)	0.340
EIM	Y	−0.14 (−3.63–3.35)	0.939
Perianal disease	Y	−0.83 (−4.79–3.14)	0.681
severity	Y	1.05 (−2.48–4.59)	0.559
Smoking	Y	1.01 (−3.46–5.65)	0.633
